# The OECD Program to Validate the Rat Hershberger Bioassay to Screen Compounds for *in Vivo* Androgen and Antiandrogen Responses: Phase 2 Dose–Response Studies

**DOI:** 10.1289/ehp.9666

**Published:** 2007-01-17

**Authors:** William Owens, L. Earl Gray, Errol Zeiger, Michael Walker, Kanji Yamasaki, John Ashby, Elard Jacob

**Affiliations:** 1 Procter & Gamble, Cincinnati, Ohio, USA; 2 U.S. Environmental Protection Agency, Research Triangle Park, North Carolina, USA; 3 Errol Zeiger Consulting, Chapel Hill, North Carolina, USA; 4 Health Canada, Ottawa, Ontario, Canada; 5 Chemicals Evaluation and Research Institute, Oita, Japan; 6 Syngenta Central Toxicology Laboratory, Macclesfield, Cheshire, UK; 7 BASF, Ludwigshafen, Germany

**Keywords:** androgen, antiandrogen, bulbocavernosus, Cowper’s glands, DDE, endocrine disruption, Finasteride, glans penis, Hershberger, levator ani, seminal vesicles, linuron, methyl testosterone, procymidone, trenbolone, validation, ventral prostate, vinclozolin

## Abstract

**Objective:**

The Organisation for Economic Co-operation and Development (OECD) has completed phase 2 of an international program to validate the rodent Hershberger bioassay.

**Design:**

The Hershberger bioassay is designed to identify suspected androgens and antiandrogens based on changes in the weights of five androgen-responsive tissues (ventral prostate, paired seminal vesicles and coagulating glands, the levator ani and bulbocavernosus muscles, the glans penis, and paired Cowper’s or bulbourethral glands). Protocol sensitivity and reproducibility were tested using two androgen agonists (17α-methyl testosterone and 17β-trenbolone), four antagonists [procymi-done, vinclozolin, linuron, and 1,1-dichoro-2,2-bis-(*p*-chlorophenyl)ethylene (*p,p’*-DDE)], and a 5α-reductase inhibitor (finasteride). Sixteen laboratories from seven countries participated in phase 2.

**Results:**

In 40 of 41 studies, the laboratories successfully detected substance-related weight changes in one or more tissues. The one exception was with the weakest antiandrogen, linuron, in a laboratory with reduced sensitivity because of high coefficients of variation in all tissue weights. The protocols performed well under different experimental conditions (e.g., strain, diet, housing protocol, bedding, vehicle). There was good agreement and reproducibility among laboratories with regard to the lowest dose inducing significant effects on tissue weights.

**Conclusions:**

The results show that the OECD Hershberger bioassay protocol is reproducible and transferable across laboratories with androgen agonists, weak androgen antagonists, and a 5α-reductase inhibitor. The next validation phase will employ coded test substances, including positive substances and negative substances having no androgenic or antiandrogenic activity.

The Organisation for Economic Co-operation and Development (OECD) undertook the revision of existing guidelines and development of new guidelines for screening and testing of potential endocrine disruptors in 1997 (OECD 1998). Validation of new guidelines is managed by a Validation Management Group (VMG). One VMG program is the rodent Hershberger bioassay, which is intended to be used as a screen for suspected androgen agonists and antagonists, and to assist in compound prioritization for further evaluation. In the Hershberger program phase 1, standardized protocols were developed and successfully tested against the high-potency reference androgen testosterone propionate (TP) and the antiandrogen flutamide (FLU). The protocols were robust, reproducible, and transferable across laboratories using these reference compounds (Owens et al. 2006). Therefore, the VMG proceeded with the design and execution of phase 2 to demonstrate the ability of the protocol to identify weakly active androgenic and antiandrogenic substances and 5α-reductase inhibitors.

## Phase 2 Design

The goals of the Hershberger phase 2 validation were as follows:

- To evaluate the reproducibility of the protocols for identifying weaker androgen agonists and antagonists
- To evaluate the capability of the protocol to detect a 5α-reductase inhibitor
- To continue to evaluate five target accessory tissues and glands of the male reproductive tract as mandatory protocol end points
- To characterize possible sources of variability among the participating laboratories.

### Standardized protocol

The principle of the Hershberger bioassay is that organs and accessory tissues in the male reproductive tract are under the control of androgens, which are necessary to stimulate and maintain growth of these tissues; the tissue growth response is relatively rapid, allowing the assay to be conducted in a matter of days; the tissue weights are quantitative; and no specialized facilities or equipment are necessary. The phase 2 androgenic and antiandrogenic protocols were largely unchanged from phases 1A and 1B (Owens et al. 2006). The primary modifications were *a*) exclusion of dorsolateral prostate weights; and *b*) specification of castration on or after postnatal day (PND) 42, because some animals castrated before PND42 in phase 1 did not undergo preputial separation, compromising glans penis (GP) dissection. The test protocols are based on weights of five mandatory male reproductive tract tissues—ventral prostate (VP), paired seminal vesicles and coagulating glands (SVCG), the levator ani and bulbocavernosus muscles (LABC), the glans penis (GP), and the paired Cowper’s or bulbourethral glands (COWS)—after 10 consecutive days of test substance administration to castrated male rats. Optional protocol measurements included liver weight, paired adrenal weight, and paired kidney weight; laboratories were permitted to perform other measurements on a voluntary, information-gathering basis. The mandatory, optional, and investigational measurements performed by each laboratory are identified in Table 1. The full model protocol for phases 1A and 1B may be found in Section I of the Supplementary Material for Owens et al. (2006) (available online at http://www.ehponline.org/docs/2006/8751/suppl.pdf).

### Participating laboratories

Sixteen laboratories from seven nations (Denmark, France, Germany, Japan, the Republic of Korea, the United Kingdom, and the United States) participated in phase 2. All laboratories participated on a voluntary and self-supporting basis, and all had participated in phase 1 (Owens et al. 2006).

### Chemicals and selected doses

An important criterion for a validation study is the demonstration of the ability to correctly identify the outcomes in the assays to be replaced or the outcomes from the apical assays (OECD 2005). Therefore, compounds were selected that produced androgenic or anti-androgenic effects in reproductive and developmental assays. These studies also provided no observed effect level (NOEL) and lowest observed effect level (LOEL) doses for comparisons to the Hershberger bioassay data (Bowman et al 2003; Clark et al. 1990; Gray et al. 1994, 1999; Hellwig et al. 2000; Imperato-McGinley et al. 1986; Kelce et al. 1995; McIntyre et al. 2000, 2002a, 2002b; Monosson et al. 1999; Ostby et al. 1999; Wilson et al. 2002; You et al. 1998).

Single chemical lots were purchased by the European Chemical Industry Council and Japan Chemical Industry Association and transferred to a central chemical repository at TNO, The Netherlands. The repository distributed chemicals to all laboratories with the exception of trenbolone, which is a controlled substance whose import and distribution are regulated. Trenbolone was obtained by the individual qualifying laboratories from a lot reserved by the supplier for the validation program. The reference agonist was testosterone propionate [Chemical Abstracts Service registry no. (CASRN) 57-85-2, 99.9% pure; Sigma-Aldrich, St. Louis MO, USA), and the reference antagonist was flutamide (CASRN 13311-84-7, > 99% pure; Sigma-Aldrich). The test substances were 17α-methyltestosterone [MT; CASRN 58-18-4, 99.6% pure; Fluka, Buchs, Switzerland), vinclozolin [VIN; 3-(3,5-dichlorophenyl)-5-methyl-5-vinyl-1,3-oxazolidine-2,4-dione; CASRN 50471-44-8, 99.2% pure; BASF AG, Germany], procymidone {PRO; 3-(3,5-dichlorophenyl)-1,5-dimethyl-3-azabicyclo[3.1.0]hexane-2,4-dion; CASRN 32809-16-8, 99.9% pure; Riedel-de Haën, Germany}, linuron [LIN; 3-(3,4-dichloro-phenyl)-1-methoxy-1-methylurea); CASRN 330-55-2, 99% pure; Crescent Chemical Co. Inc., Islandia, NY, USA), 17β-trenbolone [TREN; 17β-hydroxyestra-4,9,11-trien-3-one; CASRN 10161-33-8, 96.6% pure; Sigma-Aldrich), *p,p’*-DDE [DDE; 1,1-dichoro-2,2-bis-(*p*-chlorophenyl)ethylene); CASRN 72-55-9, 99.5% pure; Sigma-Aldrich), and finasteride [FIN; CASRN 98319-26-7, 99.7% pure; Apin Chemicals Ltd., Abingdon, Oxfordshire, UK).

The dose series for each substance was specified in order to compare results and assess test reproducibility among the laboratories. To assess interlaboratory variability, each substance was tested in at least three laboratories. Because of budget concerns, seven laboratories began testing approximately 1 year before the others; these tests were designated stage 1. In stage 1, the participating laboratories used a stimulating TP dose of 0.2 mg/kg body weight (bw)/day for the antagonist studies. In stage 2, the other laboratories used a stimulating TP dose of 0.4 mg/kg bw/day. A total of 41 separate studies were performed. The doses tested are described in Table 2.

All substances were prepared in corn oil based on repository instructions, with the exception of laboratory 2, which used methyl cellulose; the dose volumes to be administered were calculated based on daily body weights in order to maintain selected doses. All the doses were administered for 10 consecutive days at approximately 24-hr intervals. The animals were sacrificed approximately 24 hr after the final dose was administered.

### Animals and husbandry conditions

Participating laboratories obtained animals from their normal external or internal sources, and recorded the strain and animal supply sources. All studies were performed in accordance with the OECD’s guidelines on animal care (OECD 2000) and appropriate national regulations. The specified husbandry conditions were the same as in phase 1 (Owens et al. 2006), and the actual parameters for each laboratory are presented in Table 3.

### Study management and quality control

The laboratories were asked to perform the studies under the OECD Good Laboratory Practice guidelines (OECD 2002) and most, but not all, did so. After the data were assembled and an initial statistical analysis performed, all laboratories were requested to audit their raw data and to respond to specific queries on outliers and questionable values. A small number of data corrections were made as a result.

### Data reporting and statistical analyses

Similar to phase 1, each participating laboratory received a standardized Excel spreadsheet (Microsoft, Redmond, WA, USA) for recording and transmitting data for analysis (Owens et al. 2006). Data entered in the spreadsheet included names and assigned duties of laboratory personnel; parameters such as rat strain, diet, and bedding with suppliers and lots; dates of castration and the initiation of treatment; caging practices; the procedures used to randomize animals into groups; individual animal numbers, daily body weights, preputial separation observations times of administration, administration volumes; clinical signs; and all mandatory and optional end points measured.

The Lead Laboratory [U.S. Environmental Protection Agency (EPA); laboratory of L.E.G.] performed its series of statistical calculations using PROC MEANS and PROC GLM in SAS (version 6.08; SAS Institute, Cary, NC, USA) based on an analysis of covariance (ANCOVA) *F*-test, followed by a pairwise *t*-test comparison between a group control and a test substance group (Owens et al. 2006). The OECD Secretariat conducted additional statistical analyses for the mandatory end points using S-Plus (Insightful Corp., Seattle, WA, USA) based on Dunnett’s multiple comparison procedure for multiple pairwise comparisons. Both starting and terminal body weights were used in an ANCOVA adjustment (Owens et al. 2006). Because group number influences the Dunnett’s error term, positive TP controls, if performed, were excluded in the agonist series, and vehicle and positive FLU controls were excluded from the antagonist and 5α-reductase series. Outliers were observed in a few data sets (defined as Studentized Residuals > 4 or < –4), but these outliers were included in all of the statistical analyses results shown here. *R*^2^ values for overall correlations and for different effects (e.g., chemical and laboratory effects) were calculated as reported previously (Owens et al. 2006) to assess the robustness of the dose response for each tissue.

The primary difference between the two statistical approaches is that pairwise *t*-test is slightly more liberal in achieving statistical significance. That is, single pairwise comparisons may achieve statistical significance in some marginal cases where Dunnett’s multiple comparisons do not. The results of both analyses are reported, side-by-side, for the mandatory end points.

Using benchmark dose (BMD) methodology, the results for each tissue were also compared for both the individual laboratory and the pooled data from all laboratories using the same test substance and, where applicable, the same stimulating TP dose. In these studies, a “hybrid” model was fit whereby the probability of being abnormal was described using a Weibul distribution (Crump 1995). In this case, “abnormal” was defined by the 5th percentile of the control distribution in the direction of adverse response (lower percentile for a decreasing adverse response, and upper percentile for increasing adverse response). The BMD was defined as the dose that increases the risk or probability of being “abnormal” by 5% over background. The lower 95% confidence limit of the BMD (BMDL) was also calculated. The program BENCH_C was used for all BMD calculations (Crump and Van Landingham 1996).

## Results

### Phase 2, Stages 1 and 2: Androgen Agonist Dose Responses

Two androgen agonists were employed as test substances in phase 2. MT was tested in eight laboratories using two overlapping dose series. TREN was tested in three laboratories. All laboratories completed their assigned studies and provided the Excel spread sheets containing all individual animal results and protocol descriptions. For the agonist studies, the means, SDs, and statistical results for the starting and terminal body weights, all five mandatory tissues, and measured optional organs from each laboratory are available in the Supplemental Material (http://www.ehponline.org/docs/2007/9666/suppl.pdf).

#### Methyl testosterone

**Figure 1:**
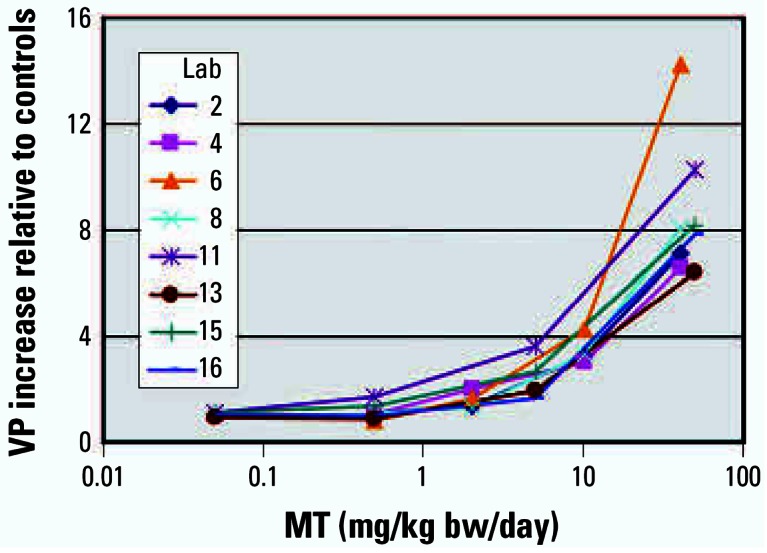
Relative increases in VP mean weights with MT administration in eight laboratories (Lab) using two dose series (laboratories 2–8 used 0.5, 2, 10, and 40 mg/kg bw/day MT, and laboratories 11–16 used 0.05, 0.5, 5, and 50 mg/kg bw/day MT).

The detection of androgen agonists was assessed in stage 1 by four laboratories using doses of 0.05, 0.5, 5, and 50 mg MT/kg bw/day and, in stage 2, by four laboratories using doses of 0.5, 2, 10, and 40 mg MT/kg bw/day. All five male sex accessory tissues responded to MT in a dose-responsive manner, and all tissues achieved statistically significant weight increases in all laboratories. The stage 1 MT studies were conducted approximately 1 year before the stage 2 MT studies. The tissue responses across both stages were reproducible based on the increases in tissue weights relative to the vehicle control from the eight individual laboratories, as illustrated by the VP results (Figure 1). In laboratory 6, absolute VP weights were less than half and absolute COWS weights were only about one-sixth those in other laboratories, suggesting differences in the dissection and tissue handling techniques.

When the stage 1 data were pooled across the participating laboratories, all five mandatory end points achieved statistical significance using the pairwise comparison approach from 5 mg MT/kg bw/day. The *R*^2^ analyses indicated a strong overall chemical relationship and a strong dose relationship, and suggested a slight relationship for possible laboratory effects for the GP. In those laboratories measuring optional tissues, there were significant increases in the liver weights and significant decreases in the adrenal weights at 50 mg MT/kg bw/day (data not shown).

When the stage 2 data were pooled across the four participating laboratories, the VP, LABC, and COWS achieved statistical significance using the pairwise comparison approach from 2 mg MT/kg bw/day, and SVCG and GP from 10 mg MT/kg bw/day. The *R*^2^ analyses indicated a strong overall chemical relationship and a strong dose relationship, and suggested a slight relationship for possible laboratory effects for the VP, LABC, and COWS. There were no evident changes in body weights with increasing MT doses. In the laboratories measuring optional tissues, there were significant increases in the liver weights in laboratories 2, 4, and 8 at 40 mg MT/kg bw/day, significant decreases in the adrenal weights in laboratories 2, 4, and 6 at 40 mg/kg bw/day MT, and significant increases in paired kidney weights in laboratories 4 and 8 from 10 mg MT/kg bw/day and in laboratory 6 at 40 mg MT/kg bw/day (data not shown).

#### Trenbolone

Three laboratories assessed the detection of androgen agonists using doses of 0.3, 1.5, 8, and 40 mg TREN/kg bw/day. TREN induced dose-related weight increases in all five male sex accessory tissues, and all five responses achieved statistical significance with the pairwise *t*-test approach. In laboratories 1 and 3, the VP and the SVCG were marginally significant with either, but not both, the starting or terminal body weight adjustments at 40 mg/kg bw/day using the Dunnett’s multiple comparisons approach. Within individual laboratories, the coefficients of variation (CVs) were higher with the VP (29–51%), SVCG (31–37%), and COWS (22–44%), again suggesting that variations in dissection and tissue-handling proficiency had an impact on the achievement of statistical significance.

When the data were pooled across laboratories, all five mandatory end points achieved statistical significance at 40 mg TREN/kg bw/day using the pairwise comparison approach. The absolute body weight gains during the treatment period were reduced at the top two doses of TREN, and were statistically significant at 40 mg/kg bw/day TREN in laboratories 1 and 7 (*p* < 0.05) and when the data were pooled (*p* < 0.01; data not shown). Liver, paired adrenal, and paired kidney weights, were not consistently or significantly affected by TREN administration (data not shown).

### Phase 2: Antiandrogen Dose Responses with Antagonists and a 5α-Reductase Inhibitor

Four androgen antagonists and a 5α-reductase inhibitor were used as test substances. Eight laboratories conducted studies with VIN and DDE. The VIN doses were identical in all laboratories, and there were two sets of overlapping doses with DDE. PRO, LIN, and FIN studies were conducted by four laboratories. Laboratory 8, with a newly trained technician, encountered several animal deaths due to gav-age errors in an initial study with PRO, and this laboratory voluntarily performed a second study; both sets of data were included in the overall analyses. For the antagonist studies, the means, SDs, and statistical results for the starting and terminal body weights, all five mandatory tissues, and optional organs from each laboratory are available in the Supplemental Material (http://www.ehponline.org/docs/2007/9666/suppl.pdf).

#### Vinclozolin

**Figure 2:**
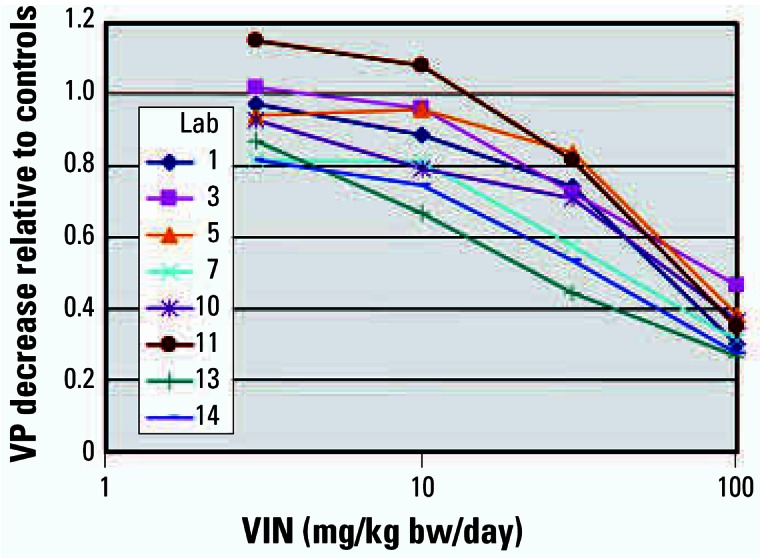
Relative decreases in VP mean weights using VIN against TP controls in eight laboratories (Lab). Laboratories 1–7 used a stimulating dose of 0.4 mg/kg bw/day TP, and laboratories 10–14 used a stimulating dose of 0.2 mg/kg bw/day TP.

Eight laboratories tested the antagonistic effects of VIN using doses of 3, 10, 30, and 100 mg/kg bw/day. In stage 1, four laboratories coadministered TP at 0.2 mg/kg bw/day, and in stage 2, four laboratories coadministered TP at 0.4 mg/kg bw/day. The stage 1 studies were conducted approximately 1 year before the stage 2 studies. The tissue responses were reproducible within and across stages 1 and 2 and were consistent based on the decreases in TP-stimulated tissue weights relative to the TP-stimulated control from the eight individual laboratories, as illustrated by the VP results (Figure 2).

VIN induced dose-responsive, statistically significant decreases in all TP-stimulated tissues with one exception. In laboratory 5, the GP was dissected only where preputial separation had occurred, reducing the high-dose group to only two animals. The absolute GP tissue weight decrease was similar to that seen in the other laboratories, but statistical significance was not achieved because of the small number of samples. When the data were pooled across the participating laboratories, all five mandatory end points achieved statistical significance using the pairwise comparison approach from 10 mg VIN/kg bw/day when using 0.2 mg TP/kg bw/day, and although the GP response was marginally insignificant, the other four mandatory end points achieved significance from 30 mg VIN/kg bw/day when using 0.4 mg TP/kg bw/day. The absolute organ weights of the liver and adrenals were statistically signifi-cantly increased by VIN administration from 30 mg/kg bw/day in all laboratories where these measurements were made (data not shown).

#### p,p’-*DDE*

**Figure 3:**
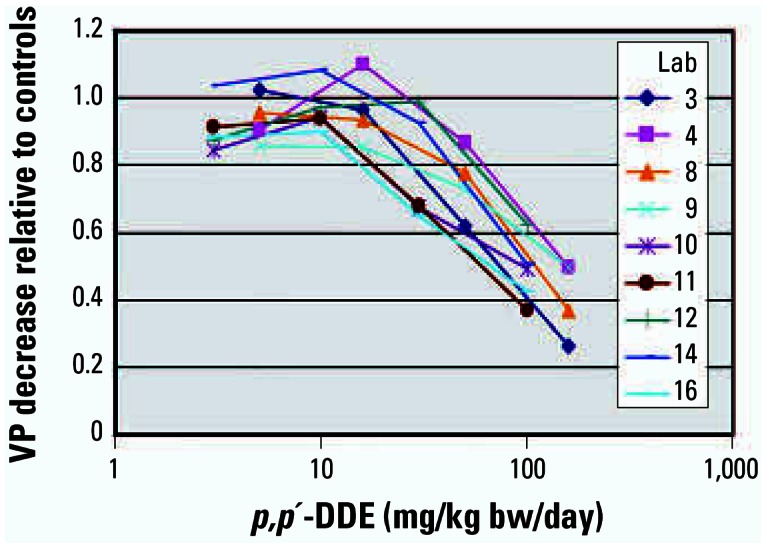
Relative decreases in VP mean weights using *p,p’*-DDE against TP controls in nine laboratories (Lab). Laboratories 3–9 used a stimulating dose of 0.4 mg/kg bw/day TP with doses of 5, 16, 50, and 160 mg/kg bw/day DDE, and laboratories 10–16 used a stimulating dose of 0.2 mg/kg bw/day TP with doses of 3, 10, 30, and 100 mg/kg bw/day DDE.

Nine laboratories assessed the detection of the weak androgen antagonist DDE. In stage 1, five laboratories used doses of 3, 10, 30, and 100 mg DDE/kg bw/day coadministered with 0.2 mg TP/kg bw/day; in stage 2, four laboratories used doses of 5, 16, 50, and 160 mg DDE/kg bw/day co-administered with 0.4 mg TP/kg bw/day. The tissue responses within and across both stages were reproducible, based on the decreases in TP-stimulated tissue weights relative to the TP-stimulated control from the eight individual laboratories, as illustrated by the VP results (Figure 3).

In stage 1, DDE induced dose-responsive, statistically significant decreases in TP-stimulated weight gains in all tissues in all laboratories. When the data were pooled across laboratories, four of the mandatory end points achieved statistical significance from 30 mg DDE/kg bw/day when using 0.2 mg TP/kg bw/day, and the GP was significant at 100 mg DDE/kg bw/day. Where measured, the liver weights were increased by DDE administration (data not shown), and there was a consistent, small, but not statistically significant decrement in terminal body weights at the high dose of 100 mg DDE/kg bw/day.

In stage 2, DDE induced dose-responsive, statistically significant decreases in TP-stimulated weight gains in all tissues in all laboratories, with two exceptions. In laboratory 4, the SVCG and the GP did not decrease in a similar absolute magnitude compared with the other laboratories. When the data were pooled across the participating laboratories, four tissues had significant decreases with both the pairwise and multiple comparison statistical approaches from 50 mg DDE/kg bw/day when using 0.4 mg TP/kg bw/day. The GP weights were significantly decreased using the pairwise approach, but statistical significance was observed in the pooled data only at 160 mg DDE/kg bw/day with Dunnett’s approach. The absolute body weights were decreased at the high dose of 100 mg/kg (data not shown), and the decrease was statistically significant in laboratories 4, 8, and 9. Liver weight increases were statistically significant in all laboratories from 16 mg DDE/kg bw/day, and the absolute increase ranged from 47 to 60% at the high dose (data not shown).

#### Procymidone

Four laboratories assessed the detection of this androgen antagonist using doses of 3, 10, 30, and 100 mg PRO/kg bw/day coadministered with 0.4 mg TP/kg bw/day.

PRO induced dose-responsive, statistically significant decreases in TP-stimulated weight gains in all five tissues in laboratories 8 and 9. In laboratories 2 and 7, the absolute GP weights decreased but did not achieve significance. When the data were pooled across laboratories, all five mandatory end points achieved statistical significance using the pairwise comparison approach from 30 mg PRO/kg bw/day, and the GP achieved significance using the Dunnett’s approach, if starting body weights were used in the ANCOVA. Body weight gains during PRO treatment were reduced by 10–20 g at the high dose, and the reductions were significant in laboratory 8 at 100 mg PRO/kg bw/day. Liver weights were significantly increased in all laboratories, and the paired adrenal weights were significantly increased in laboratories 2, 7, and 8 (data not shown).

#### Linuron

Four laboratories assessed the detection of this weak androgen antagonist using doses of 3, 10, 30, and 100 mg LIN/kg bw/day coadministered with 0.4 mg TP/kg bw/day.

LIN induced positive responses at the high dose in three of four laboratories. In laboratory 6, absolute weights of GP and COWS were largely unchanged, and the absolute decreases in VP, SVCG, and LABC weights were modest compared with values from the other laboratories. The SVCG achieved marginal significance using the *t*-test approach but was not significant with the Dunnett’s approach. Many tissue CVs in this laboratory were very high, particularly in the control group (VP 40.8%, SVCG 36.9%, COWS 64.9%), suggesting tissue dissection was a major factor in failing to detect LIN. When the data were pooled across the laboratories, four of the mandatory end points achieved statistical significance using both the pairwise comparison and Dunnett’s multiple comparison approaches at 100 mg LIN/kg bw/day. However, the GP did not achieve significance with the Dunnett’s method. Body weights were significantly decreased in laboratories 1 and 4 by LIN treatment, and absolute values decreased by 15–20 g in laboratories 5 and 6. No optional organ weight changes were attributable to LIN because weights decreased in relative proportion to body weight.

#### Finasteride

Four laboratories assessed the detection of potent 5α-reductase inhibitors using doses of 0.2, 5, 30, and 25 mg FIN/kg bw/day coadministered with 0.4 mg TP/kg bw/day.

FIN was easily detected in this protocol. A NOEL was not observed in one or more tissues at the lowest dose of 0.2 mg FIN/kg bw/day in three of the four laboratories. The absolute weights of all five sex accessory tissues decreased in a dose-responsive manner. The VP, SVCG, and COWS decreases were statistically significant in all laboratories at 25 mg FIN/kg bw/day. However, the LABC weight decrease was not consistently significant in laboratory 6, and the response of GP did not achieve significance in laboratories 2 and 6. When the data were pooled, all tissues achieved significance from 0.2 mg FIN/kg bw/day using pairwise comparisons, and all tissues, with the exception of COWS, were significant with the Dunnett’s approach from this same dose. Finasteride had no discernable impact on body weights, body weight gains, or optional organ weights (data not shown).

### Tissue LOELs with Agonist and Antagonists

The LOELs for each tissue were compared within each laboratory and across laboratories to assess the reproducibility and sensitivities of the five target tissues and the stimulating doses of 0.2 and 0.4 mg TP/kg bw/day with weak antiandrogens. The LOELs for all tissues and individual studies are available in the Supplemental Material (http://www.ehponline.org/docs/2007/9666/suppl.pdf). The large majority of LOELs for all five tissues fall between 0.5 and 1 order of magnitude for each substance across the participating laboratories. This demonstrates a high degree of reproducibility of the dose responses in the Hershberger bioassay across laboratories. As would be expected for a lower stimulating dose and constant antiandrogen dose, the LOELs were slightly lower for both VIN and DDE when 0.2 mg TP/kg bw/day was used compared with when 0.4 mg TP/kg bw/day was used.

All five sex accessory tissues achieved statistically significant LOELs using the *t*-test in 33 of a total of 41 studies. In one study (laboratory 5 with VIN), the GP was not dissected in four animals because of a lack of preputial separation. In the remaining seven studies, the GP consistently failed to achieve statistical significance, and all of these incidents were with antiandrogens. The only instance in which more than one tissue did not achieve statistical significance was the test with LIN by laboratory 6. A review of tissue sensitivity in Table 4 suggests overall equivalent sensitivity among the other four tissues (VP, SVCG, LABC, and COWS) with both androgens and antiandrogens.

### Benchmark Dose Analyses

BMDs were calculated for each mandatory tissue within each laboratory and across laboratories to compare the reproducibility and sensitivities of the five target tissues and also to compare the stimulating doses of 0.2 and 0.4 mg TP/g bw/day with weak antiandrogens. The BMDs for all tissues in the individual laboratories are reported in the Supplemental Material (http://www.ehponline.org/docs/2007/9666/suppl.pdf). Although most BMDs for a tissue were in good agreement across laboratories, there were occasional exceptions. These exceptions were attributed to one of several causes: *a*) there were only four doses available and the group size was only six animals, which introduced variation and uncertainty; *b*) in several cases, the LOEL occurred only at the highest dose and the absolute response was small, leaving only this one responsive dose in the BMD calculation, also introducing variation and uncertainty; and *c*) the TP-stimulated baseline was variable at the lower doses (i.e., the baseline means were sometimes as much as 20% higher or lower than the control, thereby introducing variability into the BMD modeling). As noted with the LOELs, the BMDs for GP were similar to the other tissues in the case of androgens, but were consistently higher in the case of antiandrogens. The BMDs, as would be expected for a lower stimulating dose and constant antiandrogen dose, were slightly lower for 0.2 mg TP/kg bw/day than for 0.4 mg TP/kg bw/day for both VIN and DDE. There were three cases, all in laboratory 6, where the BMD calculation did not indicate a dose–response relationship: the GP and COWS using LIN, and the GP using FIN.

The BMD calculations and tissue CVs for pooled data have been summarized for all five mandatory tissues and all test substances (Table 5). For agonists, no consistent differences in sensitivity among tissues were observed. For antagonists, the GP was consistently, albeit modestly, less sensitive than the other tissues. In addition, with DDE, the BMD for an increase in liver weight was less than the BMDs for any of the mandatory tissues.

## Discussion

There is a regulatory need for the Hershberger bioassay to identify and assist in the prioritization of test substances that may have androgenic or antiandrogenic mechanisms of action. Antiandrogens are a particular concern because of their effects on *in utero* male reproductive tract development. The growth responses of the Hershberger target tissues are relevant because this growth depends upon the androgen receptor (AR) and 5α-reductase activity. Further, adsorption, distribution, metabolism, and excretion interactions are sufficiently similar between the castrated male, the intact male, and the *in utero* exposures, to the Hershberger bioassay’s relevance.

This phase 2 validation was intended to test the sensitivity and reproducibility of the Hershberger protocol with androgens and anti-androgens with weaker potencies than the phase 1 reference substances, and also to test the ability of the protocol to reproducibly identify 5α-reductase inhibitors. The data support the conclusion that the OECD Hershberger assay protocol is sufficiently sensitive, robust, and reproducible to detect androgenic and antiandrogenic activities of chemicals, and can also detect 5α-reductase inhibitors. All laboratories were successful in detecting weight increases in multiple target tissues after treatment with MT and TREN, as well as weight decreases of multiple target TP-stimulated tissue weights with VIN, DDE, and FIN. Three of four laboratories testing LIN were successful; however, laboratory 6—with very high tissue CVs in both control and test substance groups—achieved marginal statistical significance in only one tissue (SVCG). Based on the CVs from this laboratory, this failure can be attributed to variability in tissue dissection and handling. As with the phase 1 validation study (Owens et al. 2006), some laboratories consistently had lower CVs, further indicating proficiency differences in dissection.

Despite differences in absolute body weights, the percentage responses of the tissues relative to the controls was very similar as shown in Figures 1, 2, and 3. Six animals per dose group were sufficient to detect the androgenic and antiandrogenic activities of these compounds. The ability of the OECD Hershberger bioassay protocol to detect these androgenic changes was not affected by differences in rat strain, diet, caging, routine laboratory procedures, or modest differences in the ages at which the animals were castrated. As with the phase 1 study, no added value could be attributed to several other measurements, for example, weights of fixed tissue and serum hormone (T or LH) analyses. Liver, kidney, and adrenal weights did provide supporting information on some chemicals and treatment conditions (e.g., liver weight increases with DDE).

The utility and sensitivity of the five mandatory sex accessory tissues were also evaluated using calculated LOELs and BMDs. There was good agreement among the individual tissues across laboratories with androgens when either the LOELs or BMDs were used. With antiandrogens, the GP was slightly less responsive than the other four mandatory tissues. The results were also in good agreement for each antiandrogenic test substance across laboratories based on LOELs, and also for BMDs, taking into account the effect of variation in the baseline TP-stimulated tissue weights on the BMD modeling.

For the optional tissues, the liver response with DDE is of interest. In the parallel validation of enhancements to the 28-day repeat dose study (Test Guideline 407), liver enlargement occurred at similar doses and paralleled increases in thyroid weight and histopathology, supporting an increased hepatic metabolism and excretion of thyroid hormones (OECD 2006). This raises the possibility that *p,p’*-DDE operates through multiple modes of action, and illustrates the need for a weight-of-evidence approach that takes all available data into account.

The results of the Hershberger bioassay with these test substances have been compared with results of androgen and antiandrogen study outcomes from developmental and reproductive assays (Table 6). A prerequisite for validation is an assessment of the predictive power of assays that will replace another assay or that operate at lower tiers (OECD 2005). This comparison supports the conclusion that the Hershberger assay is of value as a screening assay to identify and prioritize substances for possible adverse effects elicited through androgenic or antiandrogenic modes of action, and supports the hypothesis that the predictions from the lower-tier Hershberger assay are valid for higher tier tests.

## Conclusion

The results show that the OECD Hershberger assay protocols are robust, reproducible, and transferable across laboratories when using a range of androgens and antiandrogens of different potencies. The next phases of the OECD validation program will test the protocol’s reproducibility over time using blinded doses of positive test substances and chemicals having no androgenic or antiandrogenic activity.

## Figures and Tables

**Table 1:** Measurements recorded by individual participating laboratories in phase 2 of the OECD Hershberger validation program.

	Laboratory (substances dosed)															
	1	2	3	4	5	6	7	8	9	10	11	12	13	14	15	16
Measurements	L,Tr,V	F,M,P	D,Tr,V	D,L,M	F,L,V	F,L,M	P,Tr,V	D,M,P	D,F,P	D,V	D,M,V	D	M,V	D,V	M	M,D
Mandatory																
VP	Y	Y	Y	Y	Y	Y	Y	Y	Y	Y	Y	Y	Y	Y	Y	Y
SVCG	Y	Y	Y	Y	Y	Y	Y	Y	Y	Y	Y	Y	Y	Y	Y	Y
LABC	Y	Y	Y	Y	Y	Y	Y	Y	Y	Y	Y	Y	Y	Y	Y	Y
GP	Y	Y	Y	Y	Y	Y	Y	Y	Y	Y	Y	Y	Y	Y	Y	Y
COWS	Y	Y	Y	Y	Y	Y	Y	Y	Y	Y	Y	Y	Y	Y	Y	Y
Optional																
Liver	Y	Y	Y	Y	Y	Y	Y	Y	Y	N	N	N	Y	N	Y	Y
Adrenals	Y	Y	Y	Y	Y	Y	Y	Y	Y	N	N	N	N	N	N	Y^a^
Kidneys	Y	Y	Y	Y	Y	Y	Y	Y	Y	N	N	Y	Y	N	N	Y
Tissue fixation^b^	Y	Y	N	Y	N	Y	Y	Y	Y	N	N	N	N	N	N	Y
Serum T and LH	N	Y	N	Y^c^	N	N	N	N	N	N	N	N	Y	N	N	N

Abbreviations: D, *p,p’*-DDE; F, finasteride; L, linuron; LH, luteinizing hormone; M, methyl testosterone; N, did not perform optional end point; P, procymidone; T, testosterone; Tr, trenbolone; V, vinclozolin; Y, performed mandatory or optional end point. Laboratory numbers were randomly assigned and do not reflect the laboratory names or countries of origin.

a Ventral prostate, seminal vesicles, Cowper’s glands, and adrenals were fixed in laboratory 16.

b Ventral prostate was fixed and reweighed after fixation.

c Hormone analyses were done for DDE only.

**Table 2:** Selected doses for phase 2 substances.

Chemical	Doses
*p,p*’-DDE	Stage 1: 3, 10, 30, and 100 mg/kg bw/day; stage 2: 5, 16, 50, and 160 mg/kg bw/day
FIN	0.2, 1, 5, and 25 mg/kg bw/day
FLU	3 mg/kg bw/day
LIN	3, 10, 30, and 100 mg/kg bw/day
MT	Stage 1: 0.05, 0.5, 5, and 50 mg/kg bw/day; stage 2: 0.5, 2, 10, and 40 mg/kg bw/day
PRO	3, 10, 30, and 100 mg/kg bw/day
TP	Stage 1: 0.2 mg/kg bw/day; stage 2: 0.4 mg/kg bw/day
TREN	0.3, 1.5, 8, and 40 mg/kg bw/day
VIN	3, 10, 30, and 100 mg/kg bw/day

**Table 3:** Laboratory parameters and conditions for phase 2 studies.

Laboratory	Rat strain	Age at castration (PND)	Acclimation time (days)	Age at necropsy (PND)	Diet	Animals per cage
1	Wistar rats, CrlGlxBrlHan:Wl	44–46	7	61–63	Provimi Kliba SA (Provimi Kliba AG, Kaiseraugst, Switzerland)	1
2	Sprague Dawley	43–45, 47	12, 10–11	64–67, 67–68	UAR, A04C-10 (Usine d'Alimentation Rationnelle, Epinay sur Orge, France)	1
3	SPF-bred Wistar HsdCpb:WU	45–46	12–13	67–69	Provimi Kliba SA (Provimi Kliba AG)	3
4	Crl CD (SD) IGS BR Sprague Dawley	43–44, 44–46	12–14	69–70	A04 C SAFE	3
5	Brl:WIST Han@Mol outbred	42–45	14–15, 18	66–70, 70–73	Proprietary^a^	3
6	Crl:CD(SD)IGS BR	42	14–15	66–67	PMI 5002 (Purina Mills, St. Louis, MO, USA)	3
7	CD (SD) IGS BR	42–45	7	59–63	RM1 (Special Diet Services, Witham, UK)	3
8	Sprague Dawley	42	8	60	PMI 5057 (Purina Mills)	3
9	Alpk:APfSD	42–43	9–10	62–63	RM1 (Special Diet Services)	3
10	Brl Han: WIST Jcl (GALAS)	41–43	7	59–61	MF (Oriental Yeast Co., Tokyo, Japan)	3
11	Crj:CD IGS (SD)^b^	40–44	8	59–63	MF (Oriental Yeast Co.)	1
12	Brl Han: WIST Jcl (GALAS)	40–42	6	57–59	MF (Oriental Yeast Co.)	3
13	Crj:CD IGS (SD)^b^	41–44	11	63–66	MF (Oriental Yeast Co.)	1
14	Crj:CD IGS (SD)^b^	43–46	7	61–64	MF (Oriental Yeast Co.)	2
15	Crj:CD IGS (SD)^b^	41–43	7	59–61	MF (Oriental Yeast Co.)	3
16	Crj:CD IGS (SD) c^b^	42–44	7	60–62	MF (Oriental Yeast Co.)	2

Corn oil was used as vehicle except in laboratory 2, which used 0.5% methyl cellulose.

a Purified (semisynthetic) diet, prepared at laboratory 5.

b The animals were from different facilities in the same country.

**Table 4:** Summary of the LOEL dose performance (by the number of laboratories) of the five mandatory tissues in each phase 2 study.

Test substance	VP	SVCG	LABC	GP	COWS
Androgen agonists					
MT	8	8	8	8	8
TREN	3	3	3	3	3
Androgen antagonists					
PRO	5	5	5	4	5
VIN	8	8	8	7	8
LIN	3	4	4	1	3
DDE	9	9	9	9	9
5α-Reductase inhibitor					
FIN	4	4	4	3	4
Times a single tissue was the most sensitive end point	4	3	0	0	1
Times the tissue was equally sensitive with at least one other tissue	30	27	29	13	23
Times tissue was not statistically significant by either approach	1^a^	0	1^a^	7^a^	1^a^

a One common instance was laboratory 6 with LIN.

**Table 5:** BMDs (mg/kg bw/day) and CVs for all laboratories combined.

	BMD (BMDL); mean CV					
Chemical	VP	SVCG	LABC	GP	COWS	Liver
Androgens						
MT, stage 1	0.51 (0.37); 22.0	0.94 (0.66); 19.5	0.95 (0.67); 12.1	9.1 (2.9); 7.2	0.53 (0.39); 21.7	NE
MT, stage 2	3.0 (1.3); 40.5	4.8 (2.6); 31.3	2.0 (0.69); 16.4	1.8 (0.89); 18.1	1.7 (0.65); 33.4	NE
TREN	8.8 (2.2); 34.2	13.8 (4.1); 33.2	5.1 (1.5); 17.7	7.7 (3.2); 10.3	15.9 (5.3); 30.5	NE
Antiandrogens						
PRO (0.4 mg TP/kg bw/day)	1.5 (1.1); 22.3	2.2 (1.4); 19.8	5.9 (3.8); 15.2	17.8 (10.0); 10.9	3.7 (2.5); 22.5	NE
VIN (0.2 mg TP/kg bw/day)	2.3 (1.2); 19.5	2.7 (1.2); 18.8	4.6 (1.9); 11.2	7.6 (2.7); 7.4	3.6 (1.8); 19.4	NE
VIN (0.4 mg TP/kg bw/day)	9.2 (3.1); 24.6	12.2 (5.1); 20.0	8.7 (4.0); 12.6	36.0 (9.8); 10.7	35.1 (11.5); 22.9	NE
LIN (0.4 mg TP/kg bw/day)	38.3 (19.1); 25.6	22.0 (8.2); 21.7	31.3 (10.6); 12.2	100 (48.1); 12.0	44.8 (15.6); 25.3	NE
*p;p’*-DDE (0.2 mg TP/kg bw/day)	16.1 (6.2); 21.9	12.5 (5.4); 18.5	13.0 (5.1); 11.0	95.6 (71.5); 8.5	26.5 (11.3); 19.2	2.2 (0.71); 7.1
*p;p’*-DDE (0.4 mg TP/kg bw/day)	23.6 (10.1); 23.7	22.1 (10.8); 22.0	17.9 (5.7); 11.9	54.2 (21.7); 10.5	11.8 (5.8); 18.4	3.9 (2.7); 8.8
5α-Reductase inhibitor						
FIN (0.4 mg TP/kg bw/day)	0.87 (0.57); 28.7	1.4 (0.85); 25.8	8.8 (4.0); 11.9	20.6 (6.8); 9.8	0.77 (0.51); 29.2	NE

NE, no dose–response effect, so no BMD could be calculated.

**Table 6:** Comparisons of LOELs from Hershberger target tissues in validation phase 2 with those from developmental and reproductive studies using the same test substances.

Chemical	Hershberger tissue LOELs	Developmental and/or reproductive bioassay LOELs
TREN	VP: 8–40 mg/kg bw/day	Wilson et al. (2002) (subcutaneous administration)
	SVCG: 40 mg/kg bw/day	↑ Female anogenital distance ≥ 0.5 mg/kg bw/day
	LABC: 8–40 mg/kg bw/day	↓ Female areolas retention ≥ 2 mg/kg bw/day
	GP: 8–40 mg/kg bw/day	↓ Female nipple retention ≥ 0.5 mg/kg bw/day
	COWS: 40 mg/kg bw/day	No frank malformations observed in females
VIN	VP: 10–100 mg/kg bw/day	Gray et al. (1994); Monosson et al. (1999)
	SVCG: 10–30 mg/kg bw/day	Anogenital distance ↓ ≥ 3.125 mg/kg bw/day^a^
	LABC: 10–100 mg/kg bw/day	Nipple retention ↑ PND14 males ≥ 50 mg/kg bw/day
	GP: 10–100 mg/kg bw/day	Nipple retention ↑ in pubertal males ≥ 50 mg/kg bw/day
	COWS: 10–100 mg/kg bw/day	Malformations (hypospadias) ↑ ≥ 50 mg/kg bw/day
		↓ Adult VP weight at ≥ 50 mg/kg bw/day
		↓ Sperm count at 100 mg/kg bw/day
DDE	VP: 30–160 mg/kg bw/day	Gray et al. (1994); Kelce et al. (1995); You et al. (1998)
	SVCG: 30–160 mg/kg bw/day	Anogenital distance ↓ in LE but not SD rats 100 mg/kg bw/day
	LABC: 30–100 mg/kg bw/day	Nipple retention ↑ PND13 males 10 mg/kg bw/day SD rats; 100 mg/kg bw/day LE rats
	GP: 100–160 mg/kg bw/day	Malformations (hypospadias) low ↑ at 100 mg/kg bw/day in one study but not observed in second study
	COWS: 30–100 mg/kg bw/day	↓ Adult VP wts 200 mg/kg bw/day
PRO	VP: 10–30 mg/kg bw/day	Gray et al. (1994); Ostby et al. (1999)
	SVCG: 10–30 mg/kg bw/day	Anogenital distance ↓ ≥ 25 mg/kg bw/day
	LABC: 3–100 mg/kg bw/day	Retained nipples ↑ ≥ 50 mg/kg bw/day
	GP: ND–10 mg/kg bw/day	Malformations (hypospadias) ↑ ≥ 50 mg/kg bw/day
	COWS: 3–100 mg/kg bw/day	Decreased adult sex accessory tissue weights ≥ 100 mg/kg bw/day
		Histopath lesions ↑ in adult sex accessory tissue ≥ 50 mg/kg bw/day
LIN	VP: ND–30 mg/kg bw/day	Gray et al. (1994); McIntyre et al. (2000, 2002a, 2002b)
	SVCG: 30–100 mg/kg bw/day	Anogenital distance not statistically significant up to 50 mg/kg bw/day
	LABC: ND–30 mg/kg bw/day	Nipple retention ↑ PND13 males 50 mg/kg bw/day
	GP: ND–100 mg/kg bw/day	Nipple retention ↑ in pubertal males ≥ 50 mg/kg bw/day
	COWS: ND–100 mg/kg bw/day	Malformations (epididymis) ↑ ≥ 25 mg/kg bw/day
		↓ Adult dorsolateral prostate weights 50 mg/kg bw/day
		Histological abnormalities ↑ in male repro tract ≥ 25 mg/kg bw/day
FIN^b^	VP: 0.2–1 mg/kg bw/day	Bowman et al. (2003); Clark et al. (1990); Imperato-McGinley et al. (1986, 1992)
	SVCG: 0.2–1 mg/kg bw/day	Anogenital distance ↓ PND1 ≥ 0.01 mg/kg bw/day
	LABC: 0.2–5 mg/kg bw/day	Nipple retention ↑ PND13 males ≥ 0.01 mg/kg bw/day
	GP: ND–0.2 mg/kg bw/day	Nipple retention ↑ in adult males ≥ 0.1 mg/kg bw/day
	COWS: 0.2–5 mg/kg bw/day	Malformations ↑ in multiple tissues ≥ 10 mg/kg bw/day
		↓ Adult LABC weights ≥ 1 mg/kg bw/day
		↓ Adult VP and COWS weights ≥ 10 mg/kg bw/day

a Hellwig et al. (2000) did not observe anogenital changes at these concentrations in a multigenerational study.

b The lowest dose used in the validation study was 0.2 mg/kg bw/day; the data of Bowman et al. (2003) were not available when the doses for this study were selected.
